# Early cholecystectomy for recurrent *versus* first-time cholecystitis: nationwide population-based study

**DOI:** 10.1093/bjsopen/zraf166

**Published:** 2026-02-12

**Authors:** Magnus Edblom, Lars Enochsson, Hanna Nyström, Gabriel Sandblom, Urban Arnelo, Oskar Hemmingsson, Ioannis Gkekas

**Affiliations:** Department of Diagnostics and Intervention, Surgery, Umeå University, Umeå, Sweden; Department of Diagnostics and Intervention, Surgery, Umeå University, Umeå, Sweden; Division of Orthopaedics and Biotechnology, Department of Clinical Science, Intervention, and Technology (CLINTEC), Karolinska Institutet, Stockholm, Sweden; Department of Diagnostics and Intervention, Surgery, Umeå University, Umeå, Sweden; Wallenberg Centre for Molecular Medicine, Umeå University, Umeå, Sweden; Department of Clinical Science and Education, Karolinska Institutet, Södersjukhuset, Stockholm, Sweden; Department of Surgery, Södersjukhuset, Stockholm, Sweden; Department of Diagnostics and Intervention, Surgery, Umeå University, Umeå, Sweden; Department of Diagnostics and Intervention, Surgery, Umeå University, Umeå, Sweden; Wallenberg Centre for Molecular Medicine, Umeå University, Umeå, Sweden; Department of Diagnostics and Intervention, Surgery, Umeå University, Umeå, Sweden

**Keywords:** acute cholecystitis, laparoscopic surgery, surgical complications, bile duct injury

## Abstract

**Background:**

Acute cholecystitis is a common complication of gallstone disease. Although early laparoscopic cholecystectomy is recommended, some patients do not undergo early surgery and remain at risk of recurrent disease. This study investigated whether early cholecystectomy for recurrent cholecystitis is associated with higher complication rates *versus* first-time cholecystitis.

**Methods:**

A retrospective population-based cohort study was conducted using data from the Swedish Registry of Gallstone Surgery. Patients undergoing early cholecystectomy for acute cholecystitis in Sweden between 1 January 2006, and 31 December 2020, were included. Patients with recurrent cholecystitis were compared to those with a first episode. The primary outcome was the total 30-day complication rate. Secondary outcomes included open surgery, prolonged surgery (≥ 120 minutes), bile duct injury, and specific complications such as intestinal injury, bleeding, reoperation, abscess, and 30-day mortality. Multivariable logistic regression was used to calculate odds ratios (OR), adjusting for age, sex, American Society of Anesthesiologists (ASA) grade, and time from admission to surgery as confounders.

**Results:**

Among 34 925 patients, 3384 had recurrent cholecystitis and 31 541 had first-time cholecystitis. The recurrent cholecystitis group had a higher complication rate (20.2 *versus* 13.8%) and an increased risk of bile duct injury (OR 2.44; 95% confidence interval (c.i.) 1.67 to 3.56), intestinal perforation (OR 2.54; 95% c.i. 1.51 to 4.25), prolonged surgery (OR 1.64; 95% c.i. 1.53 to 1.67), and open surgery (OR 1.76; 95% c.i. 1.64 to 1.92). However, patients with recurrent cholecystitis were older and had a higher ASA grade.

**Conclusion:**

Early cholecystectomy for recurrent cholecystitis is associated with increased complication rates compared with first-time cholecystitis. These findings support early surgical intervention during the first episode to reduce the risk of adverse outcomes associated with recurrent disease.

## Teaser

This population-based cohort study from Sweden found that patients undergoing early cholecystectomy for recurrent cholecystitis had higher rates of complications than those operated on during their first episode. Those with recurrent cholecystitis had increased risks of bile duct injury, intestinal perforation, prolonged surgery, and conversion to an open procedure. The findings support early surgical intervention during the initial episode to reduce the risk of adverse outcomes associated with recurrence.

## Introduction

Gallstone disease is highly prevalent in most Western countries, and its incidence increases with age. Acute cholecystitis is the most common complication of gallstone-related disease and often requires surgical treatment^1^. Approximately 20–40% of individuals with gallstones develop complications, with an annual incidence of 1–3%^2^. In approximately 10–15% of patients, the first clinical presentation is acute cholecystitis^2^. The optimal timing of surgery has historically been a matter of debate. However, current guidelines^2,3^ recommend surgical treatment, preferably early laparoscopic cholecystectomy. Several studies^4–6^ have compared early *versus* delayed surgery in the elective setting. A meta-analysis^3^ conducted during the update of the Tokyo Guidelines 2018 showed no difference in complications or mortality, but reported shorter total hospital stay and lower costs with early surgery. However, some studies^7^ have also reported lower complication rates with early cholecystectomy.

Despite increasing evidence and current recommendations, a large number of patients do not undergo early cholecystectomy during initial admission for acute cholecystitis^8,9^. These patients remain at risk of recurrent gallstone-related complications. Evidence on recurrence rates of gallstone complications after acute cholecystitis is limited, but a meta-analysis^10^ revealed that 22% of patients treated without surgery (conservatively) experienced relapse of gallstone-related disease. A long-term follow up study^11^ from the pre-ultrasound era reported a recurrence of acute cholecystitis after the first episode in 10–20% of patients, with most cases of recurrence occurring within 1 year. A more recent study^12^ have reported that 6–23% of patients awaiting elective surgery after index admission for acute cholecystitis, experience relapse of symptoms requiring emergency cholecystectomy when waiting for elective surgery. In addition, evidence suggests that 29% of patients experience a subsequent gallstone-related event (emergency department visit or hospital admission) within 1 year^9^. A Cochrane review^13^ of randomized clinical trials investigating early *versus* delayed cholecystectomy found that 18% of patients randomized to delayed cholecystectomy developed recurrent symptoms necessitating emergent cholecystectomy while waiting for elective surgery. In clinical practice, recurrent cholecystitis is frequently encountered, but data on the safety of surgery for patients with recurrent cholecystitis are limited. A retrospective cohort study^12^ of 434 patients comparing early surgery and delayed elective surgery for recurrent cholecystitis found no difference in gastric or intestinal injury, bile duct injury, postoperative collection, or mortality. To our best knowledge, no studies have compared early cholecystectomy for first-time cholecystitis to early cholecystectomy for recurrent cholecystitis.

The aim of this study was to investigate whether early cholecystectomy for recurrent cholecystitis is associated with a higher complication rate compared with early cholecystectomy for first-time cholecystitis.

## Methods

### Study design and data sources

This study was conducted as a population-based retrospective cohort study and used data from the Swedish National Register for Gallstone Surgery and Endoscopic Retrograde Cholangiopancreatography (GallRiks). All data are reported via an online form by the surgeon or theatre nurse in conjunction with the procedure. The online form includes a question about a history of previous episodes of acute cholecystitis. Independent local coordinators at each participating hospital register a 30-day follow-up formula by reviewing medical records and, if necessary, contacting the patient to minimize the risk of missing postoperative adverse events^14^.

### Population

Patients registered in GallRiks between 1 January 2006 and 31 December 2020 with acute cholecystitis as the indication for surgery were identified. The diagnosis of acute cholecystitis was registered by the surgeon based on clinical and radiological examinations and intraoperative findings. Exclusion criteria were as follows: elective cholecystectomies and cholecystectomies performed because of conditions other than acute cholecystitis; patients aged < 18 years; patients who underwent surgery more than 14 days after admission; and patients with missing data for any of the variables of age, sex, American Society of Anaesthesiologists (ASA) grade, operating time, and surgical approach.

Data from the register were collected retrospectively. Demographic and clinical data were recorded, including age, sex, ASA grade, days between admission and surgery, day of admission and departure from hospital, bile duct injury, duration of surgery, intraoperative complications (for example, bleeding, intestinal injury, common bile duct injury), and postoperative complications within 30 days (for example, abscess, pneumonia, thrombosis, surgical site infection, common bile duct injury discovered after surgery). Patients with recurrent cholecystitis were identified based on registry data, where the reporting surgeon records in the registry form whether the patient has had a previous episode of acute cholecystitis.

### Outcomes

Patients with a previous episode of acute cholecystitis were compared with the group undergoing surgery for the first episode of cholecystitis. The primary outcome was total complication rate, including all intraoperative and postoperative complications within 30 days (such as surgical complications, thrombosis, pulmonary and cardiac complications, and wound infections). Secondary outcomes were the rate of open surgeries or conversion to open surgery, prolonged surgery with an operating time ≥ 120 minutes, and the frequency of bile duct injuries. Finally, the rate of specific complications, such as intestinal injury, bleeding, reoperation, infection with abscess, and 30-day mortality, was analysed. Age, sex, ASA grade, and time from admission to surgery were considered as potential confounders and included in the multivariable analysis.

### Statistical analysis

Statistical analyses were performed using SPSS^®^ Statistics version 28.0.1.1 (IBM, Armonk, NY, USA). Patient and surgical characteristics are presented in a comparative characteristics table. *P* values were calculated using χ^2^ tests for categorical variables and two-tailed *t*-tests for continuous variables. *P* < 0.050 was considered statistically significant. Comparisons between the two groups and their risk of complications, bile duct injury, conversion to open surgery, length of hospital stay (> 3 days), and 30-day mortality were made using multivariable logistic regression analysis, adjusting for the confounding factors of age, sex, ASA grade, and time from admission to surgery. Only patients with complete data were included in the analysis. Results are presented as odds ratios (ORs) with their 95% confidence interval (c.i.) and *P* value. In these analyses, two-tailed test with *P* < 0.050 was considered significant.

### Ethical considerations

This study was approved by the Swedish Ethical Review Authorithy (Dnr 2024-07355-02). The study followed the STROBE reporting guideline^15^.

## Results

### Clinical characteristics

**Fig. 1:**
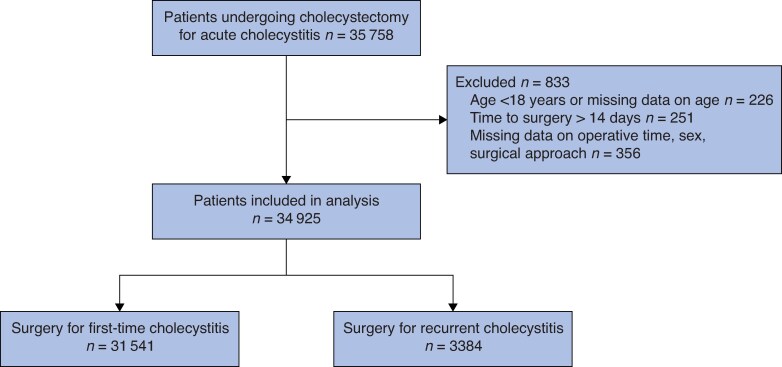
Flow chart of patient inclusion in the study and procedures performed

In all, 35 758 patients underwent surgery for acute cholecystitis and were eligible for inclusion in the study. Of these patients, 833 were excluded after applying the exclusion criteria. Thus, 34 925 patients were included in the analysis, 3384 of whom underwent surgery for recurrent cholecystitis and 31 541 who underwent surgery for first-time cholecystitis (*Fig. 1*). There were 16 782 men (48.1%) and 18 143 women (51.9%) in the study cohort, and the mean age across the cohort was 56.5 years. The demographic characteristics of the recurrent cholecystitis and first-time cholecystitis groups are presented in *Table 1*. There were some differences between the two groups, with the recurrent cholecystitis group having higher mean age (60.8 *versus* 56.0 years; *P* < 0.001), a larger proportion of patients aged >75 years (20.9 *versus* 11.9%; *P* < 0.001), and fewer patients with an ASA grade I classification (26.9 *versus* 37.1%; *P* < 0.001). The group with recurrent cholecystitis had a higher proportion of open surgeries and laparoscopic procedures converted to open surgery, longer hospital stays, and longer mean operative time (*Table 2*).

**Table 1:** Demographic characteristics of included patients

	First-time cholecystitis (*n* = 31 541)	Recurrent cholecystitis (*n* = 3384)	Difference (%)	P*
**Age (years)**				
18–50	11 659 (37.0%)	932 (27.5%)	9.5	<0.001
51–75	16 119 (51.1%)	1744 (51.5%)	−0.4	
>75	3763 (11.9%)	708 (20.9%)	−9.0	
**Sex**				
Male	14 999 (47.6%)	1783 (52.7%)	−5.1	<0.001
Female	16 542 (52.4%)	1601 (47.3%)	5.1	
**ASA grade**				
I	11 698 (37.1%)	910 (26.9%)	10.2	<0.001
II	15 465 (49.0%)	1709 (50.5%)	−1.5	
III	4119 (13.1%)	707 (20.9%)	−7.8	
IV	251 (0.8%)	55 (1.6%)	−0.8	
V	8 (0.0%)	3 (0.1%)	−0.1	

Values are *n* (%) unless otherwise stated. ASA, American Society of Anesthesiologists.*χ^2^ test were used for calculation of *P* values.

**Table 2:** Characteristics of included operations

	First-time cholecystitis (*n* = 31 541)	Recurrent cholecystitis (*n* = 3384)	Difference (%)	P*
**Surgical approach**				
Laparoscopic	23 867 (75.7%)	2029 (60.0%)	15.7	<0.001
Laparoscopic, converted to open	3971 (12.6%)	753 (22.2%)	−9.7	
Open	3703 (11.7%)	602 (17.8%)	−6.1	
**Time from admission (days)**				
0	3901 (12.4%)	393 (11.6%)	0.8	<0.001
1	12 758 (40.4%)	1123 (33.2%)	7.2	
2	8478 (26.9%)	869 (25.4%)	1.5	
3	3580 (11.3%)	442 (13.1%)	−1.8	
4	1455 (4.6%)	236 (7.0%)	−2.4	
≥5	1369 (4.3%)	321 (9.5%)	−5.2	
LOS† (days), mean(s.d.)	4.9(7.1)	6.6(8.7)		<0.001^a^
Operating time (min), mean(s.d.)	118(54)	136(61)		<0.001^a^

Values are *n* (%) unless otherwise stated. †Excluded because of missing data: recurrent cholecystitis, 133; first-time cholecystitis, 710. LOS, length of hospital stay; s.d., standard deviation; min, minutes. *χ^2^ test were used for calculation of *P* values. ^a^ Two-sided *t*-test were used for calculation of *P* values.

### Complications

There were differences in complication rates between the two groups, with a total complication rate of 20.2% in the recurrent cholecystitis group compared with 13.8% in the first-time cholecystitis group. After adjusting for the confounding factors of age, sex, ASA grade, and time from admission to surgery, patients with recurrent cholecystitis were found to have an OR for total complications of 1.38 (95% c.i. 1.26 to 1.52; *P* < 0.001). The group with recurrent cholecystitis also had higher rates and ORs for intraoperative complications and 30-day complication rates (*Table 3*).

**Table 3:** Multivariable logistic regression for different outcomes after surgery

	No. of patients (%)	Multivariable analysis*	
		Odds ratio	*P*
**Total complication rate**†			
First-time cholecystitis	30 815 (13.8%)	1.00 (reference)	<0.001
Recurrent cholecystitis	3308 (20.2%)	1.38 (1.26, 1.52)	
**Intraoperative complication rate**‡			
First-time cholecystitis	31 536 (2.9%)	1.00 (reference)	<0.001
Recurrent cholecystitis	3384 (5.2%)	1.61 (1.36, 1.90)	
**30-day complication rate§**			
First-time cholecystitis	30 819 (11.7%)	1.00 (reference)	<0.001
Recurrent cholecystitis	3308 (16.7%)	1.31 (1.18, 1.45)	
**Operation completed as open surgery**			
First-time cholecystitis	31 541 (24.3%)	1.00 (reference)	<0.001
Recurrent cholecystitis	3384 (40.0%)	1.76 (1.64, 1.92)	
**Operative time ≥ 120 min**			
First-time cholecystitis	31 541 (43.4%)	1.00 (reference)	<0.001
Recurrent cholecystitis	3384 (56.6%)	1.64 (1.53, 1.67)	
**LOS > 3 days**¶			
First-time cholecystitis	30 831 (52.7%)	1.00 (reference)	<0.001
Recurrent cholecystitis	3251 (68.1%)	1.61 (1.46, 1.77)	

*Values in parentheses are 95% confidence intervals. Multivariable analyses were adjusted for patient age, sex, American Society of Anesthesiologists grade, and time from admission to surgery. †Excluded because of missing data: first-time cholecystitis, 726; recurrent cholecystitis, 76. ‡Excluded because of missing data: first-time cholecystitis, 5. §Excluded because of missing data: first-time cholecystitis, 722; recurrent cholecystitis, 76. ¶Excluded because of missing data: first-time cholecystitis, 710; recurrent cholecystitis, 133. min, minutes; LOS, length of hospital stay.

When analysing specific adverse events, the recurrent cholecystitis group had increased risks of bile duct injury, intestinal perforation, and infection with abscess (*Table 4*). The risk for bile duct injury was more than doubled in patients with recurrent cholecystitis (OR 2.44; 95% c.i. 1.67–3.56; *P* < 0.001). The risk of intestinal perforation was also significantly increased in the group with recurrent cholecystitis (OR 2.54; 95% c.i. 1.51 to 4.25; *P* < 0.001). There were no differences regarding intraoperative bleeding, reoperation, and 30-day mortality between the two groups (*Table 4*).

**Table 4:** Multivariable logistic regression for specific adverse events

	No. of patients (%)	Multivariable analysis*	
		Odds ratio	*P*
**Bile duct injury**			
First-time cholecystitis	31 541 (0.4%)	1.00 (reference)	<0.001
Recurrent cholecystitis	3384 (1.1%)	2.44 (1.67, 3.56)	
**Intraoperative bleeding**			
First-time cholecystitis	31 541 (1.1%)	1.00 (reference)	0.052
Recurrent cholecystitis	3384 (1.9%)	1.32 (1.00, 1.73)	
**Intestinal perforation**			
First-time cholecystitis	31 541 (0.2%)	1.00 (reference)	<0.001
Recurrent cholecystitis	3384 (0.6%)	2.54 (1.51, 4.25)	
**Reoperation within 30 days**†			
First-time cholecystitis	30 817 (1.5%)	1.00 (reference)	0.731
Recurrent cholecystitis	3308 (1.8%)	1.05 (0.80, 1.38)	
**Infection with abscess (30 days)**‡			
First-time cholecystitis	30 151 (2.2%)	1.00 (reference)	0.002
Recurrent cholecystitis	3226 (3.6%)	1.39 (1.13, 1.71)	
**30-day mortality**			
First-time cholecystitis	31 541 (0.3%)	1.00 (reference)	0.222
Recurrent cholecystitis	3384 (0.4%)	0.70 (0.39, 1.24)	

*Values in parentheses are 95% confidence intervals. Multivariable analyses were adjusted for patient age, sex, American Society of Anesthesiologists grade, and time from admission to surgery. †Excluded because of missing data: first-time cholecystitis, 724; recurrent cholecystitis, 76. ‡Excluded because of missing data: first-time cholecystitis, 1390; recurrent cholecystitis, 158.

### Operations

The operating time was significantly longer for patients with recurrent cholecystitis (*Table 2*), as was the OR for prolonged surgery (≥ 120 minutes), as detailed in *Table 3*. The proportion of cholecystectomies completed as open procedures was significantly higher in the group with recurrent cholecystitis, with 17.8 and 22.2% of patients undergoing open surgery and laparoscopy converted to open surgery, respectively (adding up to a total of 40% of patients undergoing open surgery). The total time in hospital was also higher in the group with recurrent cholecystitis (*Table 2*).

## Discussion

This national cohort study showed that patients with recurrent acute cholecystitis undergoing early cholecystectomy had a significantly higher risk of complications and open surgery. They also had increased risks of bile duct injury and intestinal perforation compared with patients undergoing early cholecystectomy for first-time cholecystitis.

A previous study^12^ including 434 patients and comparing emergency to elective surgery for recurrent attacks of cholecystitis found no difference in the complication rate or frequency of open surgery. In earlier studies^6,16–18^, complication rates after early cholecystectomy for acute cholecystitis were reported to be around 11.8–14.1%. This is comparable with the rate of complications in the present study in the group undergoing surgery for first-time cholecystitis. The higher risk of complications in the group with recurrent cholecystitis indicates that earlier episodes of inflammation increase the difficulty of surgery, potentially leading to more complications. One of the most feared complications of gallbladder surgery is bile duct injury, with considerable long-term impact both in terms of clinical outcomes and quality of life^19^. Previously known risk factors for iatrogenic bile duct injury include age, acute and subacute inflammation of the gallbladder, thickening of the gallbladder wall, anatomical variations in the hepatocystic triangle, and preoperative abnormal liver function tests^20^. The results of the present study showing a significantly higher risk of bile duct injury in the group with recurrent cholecystitis suggest that recurrent inflammation should also be considered a risk factor for iatrogenic bile duct injury during cholecystectomy. For symptomatic gallstone disease, open cholecystectomy is known to be associated with a longer hospital stay, but does not differ significantly from laparoscopic surgery in terms of complications^21^. However, for acute cholecystitis, laparoscopic surgery reduces morbidity and the postoperative hospital stay compared with open surgery^22,23^. In the present study, the rate of open surgery was significantly higher in the group with recurrent cholecystitis than in the group operated on for cholecystitis for the first time. In a Cochrane review^13^, a subgroup analysis showed that among those patients who waited for delayed surgery after cholecystitis and developed recurrent symptoms necessitating emergency cholecystectomy, 45% had open surgery. That rate is comparable to the rate in the present study, and this implies that patients with recurrent cholecystitis have an increased risk of open surgery and the related morbidity.

The results of the present study further strengthen the evidence underlying current guidelines^2,3^, which advocate cholecystectomy at the first occurrence of cholecystitis, thus preventing recurrent disease and subsequent surgery associated with a higher morbidity. From a health economic perspective, early surgery at the first occasion is also beneficial, with a shorter length of hospital stay and fewer readmissions. These are factors that may be considered in the evaluation of risks and benefits of surgical management of acute cholecystitis.

There are limitations to this study, the most significant of which are the limitations associated with using a large register-based database. The data are supposed to be registered by the surgeons in direct conjunction with the procedure, but this is not always possible and may lead to recall bias. In addition, there could be errors in data registration and low validity of data. However, GallRiks has been continuously validated and has high coverage and data validity^24^. Due to shortcomings in the database, there may be a risk of residual confounding. There are variables that may be of interest and could affect the result but have not been registered, or are missing to a large extent, such as the severity of acute cholecystitis, establishment of critical view of safety^25^, and body mass index. In addition, complications in this study are reported without a severity grade according to the Clavien–Dindo classification^26^. The Clavien–Dindo classification was introduced as a variable in GallRiks during 2021, the year after this study. There is also a risk of selection bias because GallRiks only includes patients undergoing surgery. Patients who, for whatever reason, do not undergo surgery are not included in the register and thereby were not included in the present study. In the group of patients undergoing surgery for recurrent cholecystitis there may have been patients who were considered too old or frail at the index admission, but for whom the initial decision to refrain from surgery was reconsidered at the repeat admission.

Demographic analysis of the two groups showed that patients undergoing surgery for recurrent cholecystitis were older and had a higher ASA grade. Previous studies^27–29^ have shown that higher age and ASA grade are risk factors for complications after cholecystectomy. This suggests that non-surgical management does not improve a patient’s fitness for surgery; rather, additional risk factors accumulate as the patient experiences recurrent cholecystitis. To account for the demographic differences between the two groups, a multivariable logistic regression analysis was performed, in which significant differences between the two groups persisted. However, as in most register-based studies, it is not possible to fully adjust for case-mix, and consequently residual confounding may exist. Despite the limitations of register-based studies, they also offer certain advantages; for example, severe complications after cholecystectomy are rare, but the use of population-based registry data makes it possible to detect differences in rare outcomes, such as bile duct injury.

The present study was performed using a Swedish national register, and so the results are applicable to the Swedish population; they may also be generalizable to countries with similar demographics and healthcare systems. However, the results should be interpreted with caution in terms of an international perspective, particularly in countries with different healthcare structures and economic systems.

In this population-based cohort study, patients undergoing early cholecystectomy for recurrent cholecystitis had a higher risk of overall complications, including bile duct injury, intestinal perforation, and open surgery, than those undergoing early surgery for first-time cholecystitis, although the group with recurrent cholecystitis was older and had higher ASA grades. These findings support an operative strategy for first-time cholecystitis.

## Data Availability

The deidentified participant data and statistical code are available from the corresponding author upon reasonable request and after approval of the proposal by the research team.
